# Infantile Mortality

**Published:** 1904-09-24

**Authors:** 


### The Hospital.

A Journal of

The Medical Sciences and Hospital Administration.

Vol. XXXVI.--No. 939. SEPTEMBER 24, 1904.

### INFANTILE MORTALITY.

A recent article in the *Times* has put in a striking light an important aspect of the question of Infantile Mortality. It has shown that the proportion of deaths among children under one year of age, generally a trustworthy index of the sanitary condition of a population, derives a new significance when it is considered in connection with a falling birth-rate. The writer points out that the national expenditure by loss of infant life in the decade 1891–1900 was at no higher rate than in the 30 years 1841–1870, but that it was levied upon a birth-income which had fallen by 15 per cent., and is still falling. A comparison of successsive quinquennial periods brings out a still worse case; for, taking the periods 1773–77 and 1898–1902, used by Dr. Tatham in the tables supplied to the Physical Deterioration Committee, we have the same increased expenditure, and an income diminished by 20 per cent. On this basis the writer indicates that, while expenditure cannot be reduced below a certain point (for all must die at some time), shrinkage of income is theoretically unlimited, and, when once begun, does actually go on until it falls below expenditure. That has already happened in countries which have preceded us on the same road, notably in the United States, where the native stock fails to reproduce itself and tends to die out. The significance of the downward movement here has been masked, the *Times* declares, by the deceptive general death-rates; but the real test of national vitality is not the prolongation of adult life but the renewal of the stock. Viewed in this light our latter-day concern about the loss of infant life might be attributed, with no great stretch of fancy, to an unconscious instinct of national self-preservation.

We have had no opportunity of going to the sources of the figures cited in the article to which we refer; but, assuming them to be correct, it is at least certain that those of later date are of a more favourable character, that the year 1902, although included in Dr. Tatham's table, gave better results than the years preceding, and that the improvement has been maintained. In that year the deaths of infants under one year of age were in the proportion of 133 per 1,000 births, against 163, 154, and 151 respectively in the previous three years, and against an average proportion of 154 in the decennial period 1892–1901. In a further investigation of the facts, contained in a letter addressed by Dr. Tatham to the Registrar General, and published by that officer in his 65th annual report, which deals with the year 1902, we find that the infantile mortality, although manifestly tending to decline, is yet greatly larger in some localities than in others, and that it is always associated with a corresponding rate of mortality in childhood, or at least in the first quinquennium of life. Among registration areas infantile mortality ranged from 71 per 1,000 births in the county of Rutland, 82 in Westmorland, 87 in Dorset, 92 in Hertford, and 94 in Huntingdon, to 143 in Stafford, 144 in the West Riding, 146 in South Wales, 147 in Nottingham, and 150 in Lancaster. A further analysis of the facts shows that the English mortality from all causes, among infants under one year of age, in the quinquennium 1897–1901, averaged 157 per 1,000 births. Out of every 1,000 children born, 170 died in the urban counties and only 128 in the rural. "Wasting diseases" brought about a mortality of 43 in the country and 45 in the town, and diarrhœal diseases came next, with a rural mortality of 20, and an urban mortality of 41. Meningitis and convulsions were responsible for a rural mortality of 18 and an urban mortality of 21, and bronchitis was responsible for 12 and 15 respectively; so that the diarrhœal group was the only one in which a great difference between the urban and the rural mortality was recorded, and must therefore be regarded as the main cause of the heavy death-rate of the towns. If the inquiry be followed to the close of the fifth year of life, it is found that the same disparity exists, and speaking generally the mortality of young children is very much greater in towns than in the country. It is noted that diphtheria is more than twice as fatal as scarlet fever, and that both are much more destructive in towns than in the country.

There are, of course, many elements in town life which are calculated to be prejudicial to the health of young children, and from which escape is only possible by removal from the range of their influence; but, after all, these are not the main factors in the national problem with which we are called upon to deal. Those factors are improper feeding and aggregation, the former as a source of diarrhœal disease, the latter as the chief means of the diffusion of epidemics. The improper feeding is largely due to the facilities afforded by town shops for the purchase of various forms of artificial food, the aggregation is largely due to the schools. The artificial foods recommended by flaring advertisements are, of course, in many cases actively injurious, and, when not actively injurious, they seldom contain the materials necessary for proper nutrition. The influence of schools in diffusing epidemics has been conclusively shown by Mr. Shirley Murphy, and one of the first duties of authorities at once sanitary and educational should be to consider in what way this influence could be diminished.

